# Comparison of gram-negative and gram-positive hematogenous pyogenic spondylodiscitis: clinical characteristics and outcomes of treatment

**DOI:** 10.1186/s12879-016-2071-4

**Published:** 2016-12-06

**Authors:** Ching-Yu Lee, Meng-Huang Wu, Chin-Chang Cheng, Tsung-Jen Huang, Tsung-Yu Huang, Chien-Yin Lee, Jou-Chen Huang, Yen-Yao Li

**Affiliations:** 1 Department of Orthopedic Surgery, Chang Gung Memorial Hospital, No. 6, West Sec., Chia Pu Rd., PuTz, Chiayi, Taiwan; 2 Division of Infectious Diseases, Department of Internal Medicine, Chang Gung Memorial Hospital, Chiayi, Taiwan; 3 Department of Ophthalmology, Chang Gung Memorial Hospital, Chiayi, Taiwan; 4 Department of Nursing, Chang Gung University of Science and Technology, Chiayi, Taiwan; 5 College of Medicine, Chang Gung University, Taoyuan, Taiwan; 6 Department of Orthopedic Surgery, Taipei Medical University Hospital, Taipei, Taiwan; 7 Graduate Institute of Clinical Medical Sciences, College of Medicine, Chang Gung University, Taoyuan, Taiwan

**Keywords:** Gram-negative infection, Gram-positive infection, Hematogenous pyogenic spondylodiscitis

## Abstract

**Background:**

To the best of our knowledge, no study has compared gram-negative bacillary hematogenous pyogenic spondylodiscitis (GNB-HPS) with gram-positive coccal hematogenous pyogenic spondylodiscitis (GPC-HPS) regarding their clinical characteristics and outcomes.

**Methods:**

From January 2003 to January 2013, 54 patients who underwent combined antibiotic and surgical therapy in the treatment of hematogenous pyogenic spondylodiscitis were included.

**Results:**

Compared with 37 GPC-HPS patients, the 17 GNB-HPS patients were more often found to be older individuals, a history of cancer, and a previous history of symptomatic urinary tract infection. They also had a less incidence of epidural abscess formation compared with GPC-HPS patients from findings on magnetic resonance imaging (MRI). Constitutional symptoms were the primary reasons for initial physician visits in GNB-HPS patients whereas pain in the affected spinal region was the most common manifestation in GPC-HPS patients at initial visit. The clinical outcomes of GNB-HPS patients under combined surgical and antibiotic treatment were not different from those of GPC-HPS patients. In multivariate analysis, independent predicting risk factors for GNB-HPS included a malignant history and constitutional symptoms and that for GPC-HPS was epidural abscess.

**Conclusions:**

The clinical manifestations and MRI presentations of GNB-HPS were distinguishable from those of GPC-HPS.

## Background

Hematogenous pyogenic spondylodiscitis (HPS) is an infection involving the intervertebral disc and adjacent vertebrae that occurs by hematogenous spread of bacteria from a distant site. HPS is highly associated with diabetes mellitus, infective endocarditis, chronic kidney disease, cancer, immunosuppressive disorders, and intravenous drug abuse. The prevalence of HPS is more frequent in males and the elderly [1, 2]. The main causative microorganisms include gram-positive cocci (GPC), especially *Staphylococcus aureus*, which account for 40-60% of HPS patients [2, 3], and gram-negative bacilli (GNB) which constitute 15–23% of cases [2, 4]. Despite the significant incidence of HPS caused by GNB, few studies in the literature are concerned with the clinical characteristics and outcomes of hematogenous spinal infections caused by GNB [5, 6].

To the best of our knowledge, no reports have compared the clinical characteristics and outcomes of HPS caused by GNB vs. GPC. The goal of this study was to evaluate clinical presentations, past infectious histories, imaging findings, and clinical outcomes in GNB-HPS vs. GPC-HPS patients.

## Methods

### Patients

**Fig. 1:**
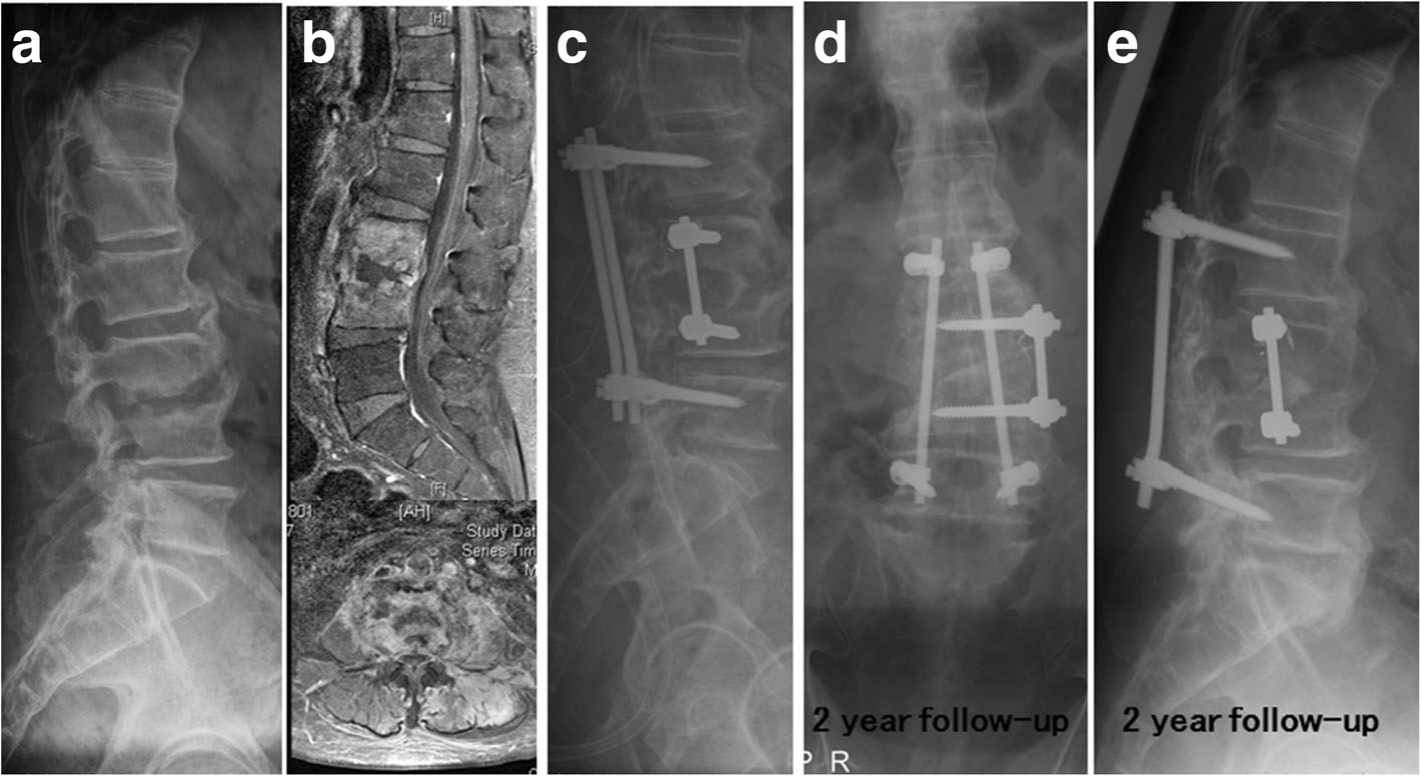
Escherichia coli infectious spondylodiscitis of L3-4 in a patient with a past history of colon cancer (**a**). Disc space was narrowing with nearby destructed endplates on L3-4 level; **b** Osteomyelitis in L3-4 vertebral bodies, discitis and psoas abscess without epidural abscess were found in gadolinium-enhanced magnetic resonance imaging; **c** Anterior and posterior spinal surgery was noticed in the immediate postoperative X-ray.; **d** and **e** Solid bone fusion was noticed at the 2-year follow-up

**Fig. 2:**
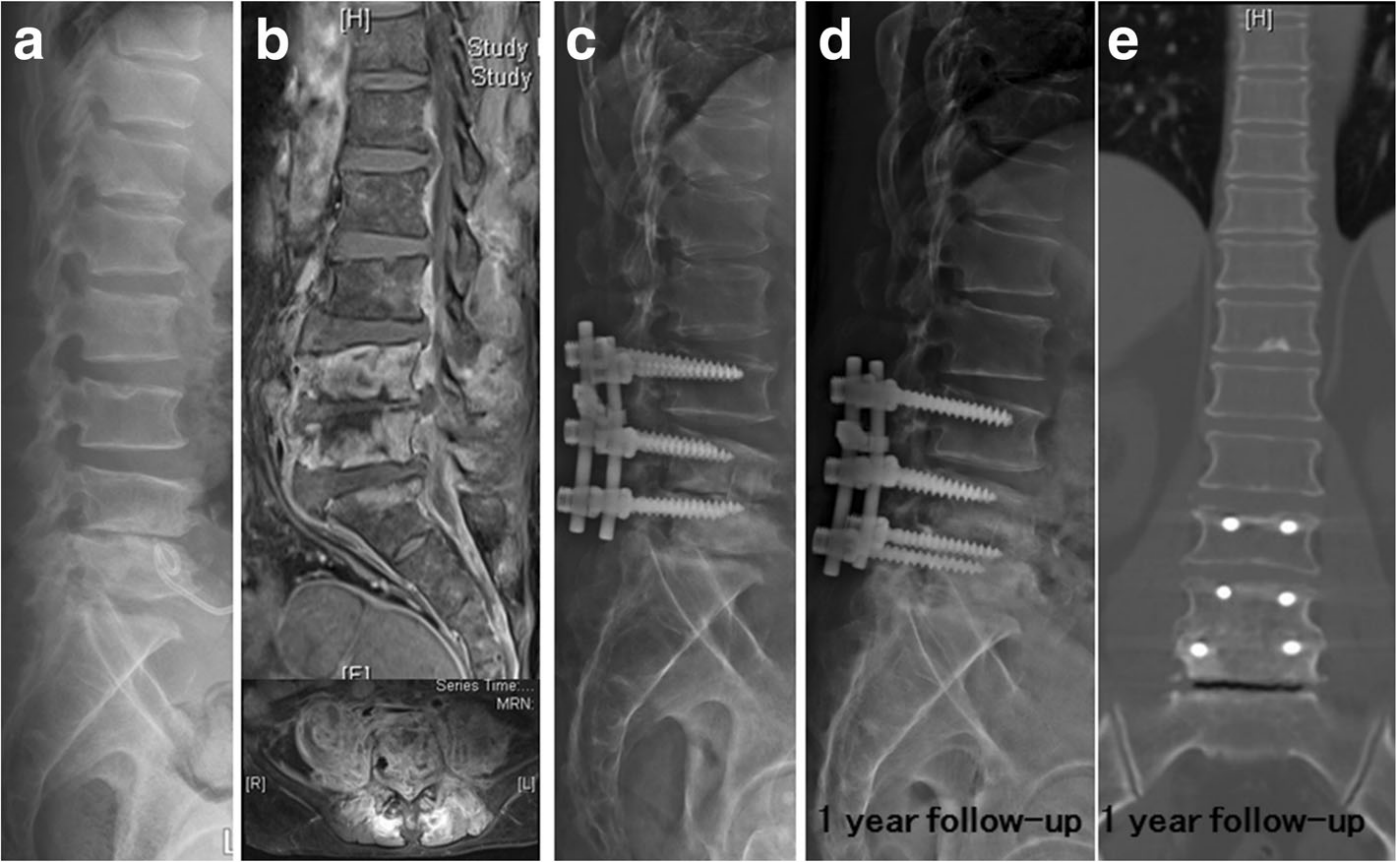
Hematogenous Pyogenic Spondylodiscitis of L4-5 due to methicillin-resistant Staphylococcus aureus infections (**a**). Narrowing disc space with endplate erosion with pigtail catheter placement for drainage of paravertebral abscess was noticed on L4-5 level; **b** Abscess formation was found in ventral epidural space, bilateral psoas and back muscles; **c** Supplementary posterior spinal instrumentation was performed 6 weeks after anterior debridement and a tricortical iliac strut bone graft for intervertebral fusion; **d** and **e** Solid bone fusion on L4-5 was noticed at the 1-year follow-up

From January 2003 to January 2013, a retrospective review of 54 patients with HPS was performed using the Spine Operation Registry of our institution. All patients included in this study underwent combined antibiotic and surgical treatment. The indications for surgical treatment included poor response to antibiotic therapy, worsening neurologic impairment, epidural abscess formation, and/or significant osseous destruction with spinal instability. Surgical approaches included anterior or posterior spinal surgery, or combined anterior and posterior approaches. Because the clinical and radiological presentations differed among the patients (Figs. 1 and 2), the surgical approach was chosen based on the individual patient’s presentation, i.e., the level of the involved vertebrae, the extent of osseous destruction, and the presence or absence of an abscess and its location, if present.

All HPS patients received a 3-month course of antibiotic therapy which consisted of at minimum 2 week course of parenteral antibiotics based on culture results. Outpatient oral antibiotic treatment completed a 3 month course of antibiotics after normalization of serum C-reactive protein (CRP) levels and leukocyte counts. Patients who survived were followed-up for a minimum of 2 years.

Pyogenic spondylodiscitis was defined as a spinal infection encompassing both vertebral osteomyelitis and discitis [7]. The definitive diagnosis of pyogenic spondylodiscitis was based on clinical presentation, imaging findings from plain radiographs and contrast-enhanced magnetic resonance imaging (MRI), and intraoperative bacteriologic cultures [4]. Exclusion criteria were: (1) negative microbiological culture from the infected specimen; (2) non-pyogenic infection; (3) surgical site infection associated with spine surgery and percutaneous spinal procedures including epidural steroid injection, selective nerve root block, and radiofrequency ablation; (4) history of previous spinal surgery on any level; and (5) history of a bedsore or penetrating wound on the back.

### Data assessment

In order to compare the clinical manifestations and outcomes between GNB-HPS and GPC-HPS patients, patient characteristics, underlying comorbidities, bacteriologic results, MRI findings, and final outcomes were reviewed using the electronic database at our hospital. The imaging findings were reviewed independently by two spine surgeons (CCC, and YYL) who were unaware of the culture results. For a history of an infectious disease, the patient sustained an infectious disease before pyogenic spondylodiscitis and received antimicrobial therapy. The history of symptomatic urinary tract infection (UTI) would be recorded when a patient was admitted for parenteral antibiotics therapy to treat cultured-proved urinary tract infection. The history of infectious diseases has been completely recorded and reviewed in every patient’s electronic chart.

A one-level spinal infection was defined as an infection involving two contiguous vertebral bodies and one adjacent intervertebral disc, and a two-level spinal infection was involving three contiguous vertebral bodies and two adjacent discs.

**Fig. 3:**
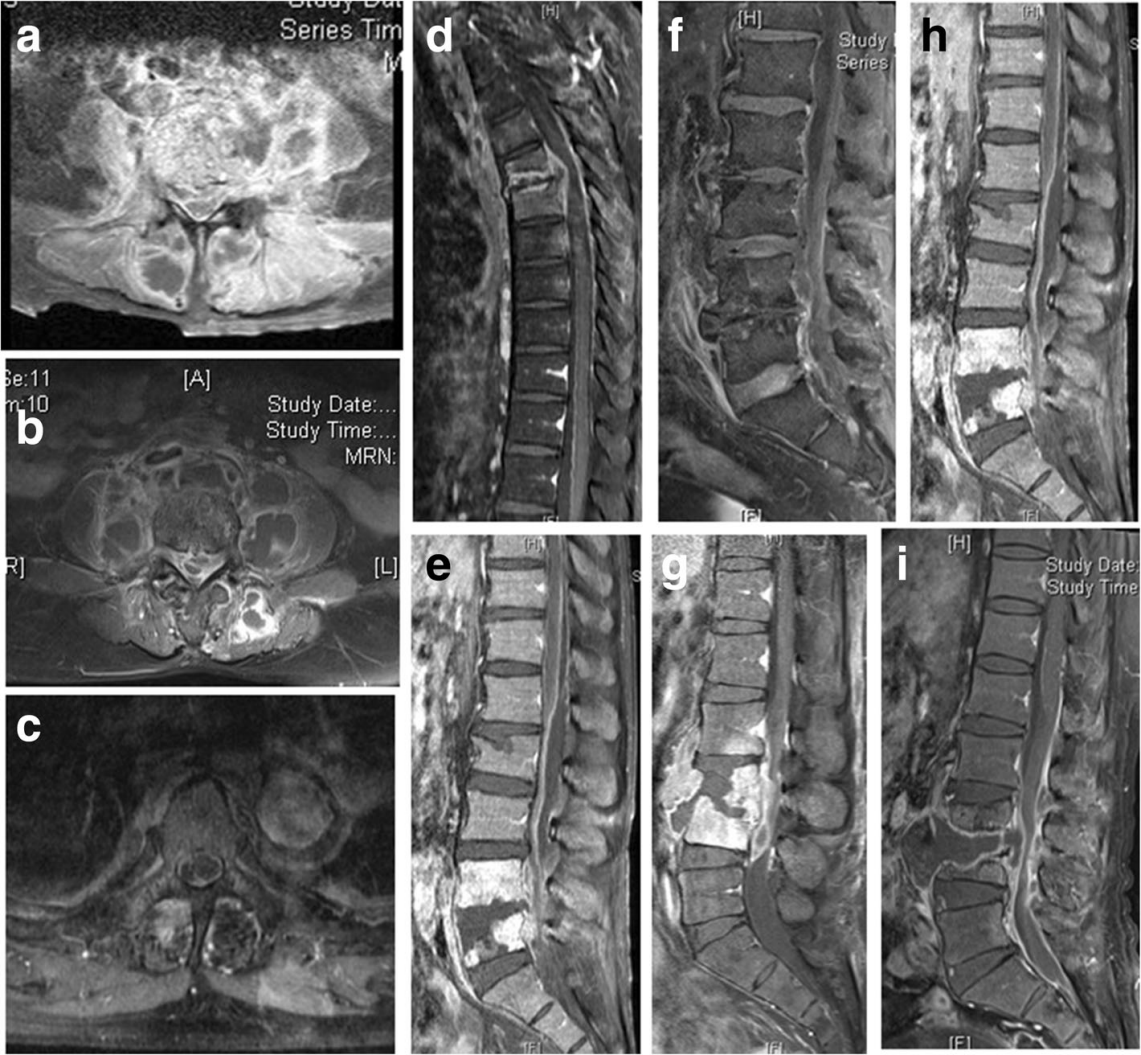
Elucidation of epidural abscess and muscle abscess in contrast-enhanced magnetic resonance imaging with fat suppression among 9 g-positive pyogenic spondylodiscitis (**a**–**c**). The spinal epidural abscess was defined as an epidural mass with iso-or hypointensity on T1-weighted images, which was surrounded by linear enhancement (ring sign) on magnetic resonance imaging. Abscess formation in psoas muscle or back muscle was defined as an asymmetrical enlarged mass of the involved muscle with ring sign on magnetic resonance imaging. **d**–**f** Ventral epidural abscess was noticed on sagittal gadolinium-enhanced fat-suppressed T1-weighted magnetic resonance

A spinal epidural abscess (Fig. 3) was defined as an epidural mass with iso-or hypointensity on T1-weighted images, hyperintensity on T2-weighted images and linear enhancement surrounding non-enhancing purulent or necrotic matter on MRI [8].

To assess clinical outcomes following combined antibiotic and surgical treatment, delayed wound healing was defined as prolonged wound exudate, or persistent fever with erythematous wound two weeks after administration of antibiotics and surgical treatment, relapsing infection was defined as recurrent spinal infection within 1 year after resolution of infection at hospital discharge. Mortality was defined as death owing to progressive sepsis or medical complications.

### Statistical methods

Univariate analysis was used to determine factors associated with GNB-HPS and GPC-HPS. An independent student *t* test was used for numerical data. A *χ*2 analysis or a Fisher’s exact test was used for categorical data. Descriptive data were presented as the mean with standard deviation for quantitative variables and as frequency for categorical variables. Statistical significance was set at a *p*-value of < 0.05. Multivariate analysis was used to determine independent predicting factors associated with GNB-HPS and GPC-HPS. All factors showing statistical significance (*p*-value of <0.05) in univariate analysis were incorporated in multivariate logistic regression analysis. Statistical analyses were performed using the Statistical Package for the Social Sciences for Windows (SPSS, version 12.0).

## Results

### Patient characteristics

A total of 54 HPS patients included 17 GNB-HPS patients and 37 GPC-HPS patients (Table 1). The average follow-up period was 4.5 years (range, 2–6 years). GNB-HPS patients were significantly older than GPC-HPS patients (mean age, 65 vs. 55 years, respectively; *p* = 0.014).

**Table 1:** Characteristics of 54patients with hematogenous pyogenic spondylodiscitis

variable	GNB (*n* = 17)	GPC (*n* = 37)	*p*
Age *(yr)*	65 ± 14.2	55 ± 14.5	0.014*
Male sex *(No. [%])*	11 (65)	31 (84)	0.162
Follow-up *(yr)*	4.8 (2–6)	4.4 (2–6)	0.372
Affected level *(No. [%])*			
1	14 (82)	28 (76)	0.732
2	3 (18)	9 (24)	
Location of spinal lesions *(No. [%])*			
Cervical spine	0	2 (5)	0.601
Thoracic spine	2 (12)	5 (14)	
Lumbosacral spine	15 (88)	30 (81)	
Symptoms at the initial visit *(No. [%])*			
Back/neck pain only	5 (30)	27 (73)	0.003*
Constitutional symptoms only	6 (35)	2 (5)	
Back pain with constitutional symptoms	6 (35)	8 (22)	
Duration of symptoms *(day)*	18 ± 21.8	37 ± 51.6	0.162
Comorbidity *(No. [%])*			
Diabetes mellitus	4 (24)	18 (49)	0.135
End stage renal failure with hemodialysis	0	5 (30)	0.168
Chronic liver disease	6 (35)	12 (32)	0.824
Malignancy	7 (41)	1 (3)	<0.001*
Intravenous drug abuse	0	6 (16)	0.161
Neurologic symptoms *(No. [%])*	12 (71)	22 (59)	0.432
Radicular pain	9 (53)	13 (35)	
Limb weakness	7 (41)	11 (30)	
Cauda equina syndrome	2 (12)	3 (8)	
Type of surgery *(No. [%])*			
Anterior surgery	6 (35)	16 (43)	0.846
Posterior surgery	7 (41)	7 (19)	
Combined surgery	4 (24)	14 (38)	
Clinical outcome *(No. [%])*			
Delayed wound healing	1 (6)	7 (19)	0.411
Relapse	1 (6)	10 (27)	0.143
Death	3 (18)	5 (14)	0.7
Radiologic result *(No. [%])*			
Solid fusion	14 (82)	32 (86)	0.696
Segmental instability	0	0	

Data are presented as the mean ± standard deviation or frequency (%), *The difference is significant (*p* < 0.05), *GNB* gram-negative bacilli, *GPC* gram-positive cocci

With regards to symptoms at initial visit, only back pain predominated in GPC-HPS (73%) compared with GNB-HPS patients (30%), and the prevalence of constitutional symptoms (fever or drowsiness) with/without back pain were more frequent in GNB-HPS patients (70% vs. 27%, *p* = 0.003). The average duration of symptoms in GNB-HPS vs. GPC-HPS patients were 18 vs. 37 days, respectively (*p* = 0.162).

Seven of 17 GNB-HPS patients had a history of malignancy, including three colorectal cancer patients, one cervical cancer patent, one lung cancer patient, one breast cancer patient, and one bladder cancer patient. One GPC-HPS patient had a history of hypopharyngeal cancer. A history of malignancy was more commonly represented in GNB-HPS compared with GPC-HPS patients (41% vs. 3%, respectively; *p* < 0.001).

Delayed wound healing was found in eight patients (one patient with GNB infection and seven patients with GPC infection); relapse of infection occurred in eleven patients (one patient with GNB infection and ten patients with GPC infection); eight deaths occurred in the HPS patients including three deaths in GNB-HPS patients and five deaths in GPC-HPS patients. Among the eight deaths, six patients died of uncontrolled infection, and two patients died of comorbidities within 3 months after hospital discharge (one from liver cirrhosis (Child-Pugh Class B) and upper gastrointestinal bleeding, and one from complicated pneumonia). All ten patients with relapsing infection had successful resolution of infection after a second surgical debridement and completion of antibiotic therapy. There were no significant differences in relapse of infection or mortality between GNB-HPS and GPC-HPS patients. All of survival patients with eradication of infection had solid fusion in spondylodiscitis at the final radiographic follow-up (*p* = 0.696).

### History of infectious disease

Twenty-three (43%) of 54 patients had histories of previous infections, including 7 (41%) GNB-HPS patients and 16 (43%) GPC-HPS patients (Table 2). GNB-HPS patients had a significantly higher frequency of previous symptomatic UTI compared with GPC-HPS patients (41% vs. 3%, respectively; *p* = 0.03). There was no significant difference in histories of other infectious disorders.

**Table 2:** The history of infectious diseases between GNB-HPS and GPC-HPS

	GNB-HPS (*n* = 17)	GPC-HPS (*n* = 37)	*p*
History of infectious disease *(No. [%])*	7 (41)	16 (43)	0.887
Musculoskeletal infection *(No. [%])*	2 (12)	11 (30)	0.189
Cellulitis	2 (12)	4 (11)	
Necrotizing fasciitis	0	5 (14)	
Osteomyelitis	0	2 (5)	
Symptomatic urinary tract infection (*No. [%])*	4 (41)	1 (3)	0.03*
Miscellaneous *(No. [%])*			
Pneumonia	1 (6)	1 (3)	
Port-A catheter infection	1 (6)	1 (3)	
Liver abscess	1 (6)	0	
Infective endocarditis	0	1 (3)	
Periorbital abscess	1 (6)	0	
AV shunt infection	0	2 (5)	

*The difference is significant (*p* < 0.05), *GNB-HPS* gram-negative bacillary hematogenous pyogenic spondylodiscitis, *GPC-HPS* gram-positive coccal hematogenous pyogenic spondylodiscitis

### Microbiologic findings

The microbiologic results from infected intraoperative specimens in the 54 HPS patients are outlined in Table 3. Among the GNB-HPS patients, *Escherichia coli* was the most commonly isolated pathogen, involved six patients (35%), followed by *Enterobacter cloacae* in four (24%) patients, *Klebsiella pneumonia* in two (12%) patients, *Salmonella enteric* in two (12%) patients, *Pseudomonas aeruginosa* in two patients (12%), *Proteus mirabilis* in one (6%) patient, and *Citrobacter freundii* in one (6%) patient. For GPC-HPS, *Staphylococcus aureus* was the most common isolate (86%). The other isolated GPC pathogens were *Coagulase-negative staphylococcus* (14%), and *Streptococcus sp*. (8%). All organisms cultured from intraoperative infected specimens were the same as those obtained from earlier blood cultures.

**Table 3:** Microbiologic findings

Microorganism	GNB (*n* = 17)
Monomicrobial *(No. [%])*	
*Escherichia coli*	5 (29)
*Enterobacter cloacae*	4 (24)
ESBL	1 (6)
*Klebsiella pneumonia*	2 (12)
*Salmonella enteric*	2 (12)
*Pseudomonas aeruginosa*	1 (6)
*Proteus mirabilis*	1 (6)
*Citrob.freundii*	1 (6)
Polymicrobial	
*Ps. aeruginosa + E.coli* (ESBL)	1 (6)
Microorganism *(No. [%])*	GPC (*n* = 37)
Monomicrobial *(No. [%])*	
*Staphylococcus aureus:*	
MSSA	18 (49)
MRSA	11 (30)
*Coag(−) staphylococcus*	2 (5)
*Streptococcus:*	
Group B *Streptococcus*	2 (5)
*Streptococcus viridans*	1 (3)
Polymicrobial	
CoNS, MSSA	1 (3)
CoNS, MRSA	2 (5)

*ESBL* Extended-spectrum β–lactamase, *MSSA* methicillin-sensitive *Staphylococcus aureus, MRSA* methicillin-resistant *Staphylococcus aureus, CoNS Coag(−) staphylococcus*

### Findings of magnetic resonance imaging

The MRI results showed that GNB-HPS patients had a significantly lower rate of epidural abscess compared with GPC-HPS patients (41% vs. 73%, *p* = 0.024) (Table 4). There were no significant differences in the incidence of psoas muscle abscess or back muscle abscess between GNB-HPS and GPC-HPS patients.

**Table 4:** Findings of magnetic resonance imaging

Variable *(No. [%])*	GNB (*n* = 17)	GPC (*n* = 37)	*p*
Back muscle abscess	6 (35)	11 (30)	0.683
Epidural abscess	7 (41)	27 (73)	0.024*
Patient number in lumbar region	*n* = 15	*n* = 30	
Psoas muscle abscess	12 (80)	21 (70)	0.755
Single involvement	3 (25)	6 (29)	
Bilateral involvement	9 (75)	15 (71)	

*The difference is significant (*p* < 0.05)

### Laboratory presentations

There was no statistically difference in laboratory parameters between two group patients, even higher average levels of CRP and erythrocyte sedimentation rates in GPC-HPS patients (*p* = 0.061 and 0.076, respectively) (Table 5). Microbial pathogens were isolated from the blood cultures of eight GNB-HPS patients and 23 GPC-HPS patients (47% vs. 62%, *p* = 0.297).

**Table 5:** Laboratory data

Variable	GNB (*n* = 17)	GPC (*n =* 37)	*p*
White blood cell count (10^3^/uL)	13.1 ± 6.3	13.8 ± 4.8	0.656
Hemoglobin (g/dL)	11.0 ± 2.4	11.7 ± 2.6	0.372
C-reactive protein (mg/dL)	110 ± 57	162 ± 95	0.061
Erythrocyte sedimentation rate (mm/h)	68.9 ± 31.2	84.3 ± 25.9	0.076
Bacteremia *(No. [%])*	8 (47)	23 (62)	0.297

### Multivariate analysis of independent predicting risk factors

Independent predicting factors for GNB-HPS were a history of cancer (OR 57, 95% CI 2–1563, *p* = 0.018), and only constitutional symptoms versus only back pain (OR 9, 95% CI 2.8–2666, *p* = 0.011) (Table 6). An independent predicting factor for GPC-HPS was epidural abscess (OR 18, 95% CI 1.4–250, *p* = 0.027).

**Table 6:** Multivariate analysis of independent risk factors

Risk factor for GNB-HPS	*p* value	OR (95% CI)
Age	0.479	
Symptoms at the initial visit	0.038*	
Constitutional symptoms only/Back pain only	0.011*	9 (2.8–2666)
Malignancy	0.018*	57 (2–1563)
UTI	0.122	
Risk factor for GPC-HPS		
Epidural abscess	0.027*	18 (1.4–250)

*The difference is significant (*p* < 0.05)

## Discussion

Our results showed that compared with the 37 GPC-HPS patients, the 17 GNB-HPS patients were more likely to be older individuals with a history of cancer. Chemotherapy/radiotherapy-induced alimentary mucositis occurs in patients treated for malignancy and the insufficient mucosal barrier contributes to enteric bacterial invasion and gram negative bacillary bacteremia with organisms such as *Escherichia coli*, *Klebsiella,* and *Pseudomonas* [9–12]. Numerous studies of bloodstream infection in the elderly indicated that gram-negative bacillary bacteremia was much more common in the elderly than in younger patients because the common infections in this population involve respiratory, urinary, and gastrointestinal systems [13–16]. A retrospective study of hematogenous vertebral osteomyelitis, conducted by Park, et al. [6], demonstrated that UTI was the main sources of infection in gram-negative vertebral osteomyelitis. Consistent with those results, age and UTI were significant factors for GNB-HPS in univariate analysis but not statistically predicting factor in multivariate analysis. The contributing reason may be that the incidence of cancer would increase with age and the high prevalence of UTI was found among the elderly.

Based on initial symptoms at first visit, this study showed that pain in the affected spinal region was the most common manifestation in GPC-HPS patients whereas constitutional symptoms (fever and drowsiness), with or without back pain, was the primary reason for initial physician visits in GNB-HPS patients. This latter finding may be attributed to be the much older age and frailty of our GNB-HPS patients. Clinical manifestations of spinal infection in aging or immunocompromised patients with cancer or poor physical function may affect their entire body with absence of localizing symptoms [17]. These patients may present with poor appetite, malaise, confusion, drowsiness, and fever. Therefore, GNB-HPS patients, who are more likely to have the early warning signs of drowsiness and fever, should seek immediate medical attention as, in contrast to patients with GPC infection, the majority of GNB-HPS patients in our study had acute onset of symptoms.

In this study, GPC-HPS possessed a higher incidence of epidural abscess formation than GNB-HPS. *Staphylococcus aureus* employs virulence factors to promote abscess formation [18, 19]. Similar to our findings, a comparative study concerning hematogenous vertebral osteomyelitis by GNB vs. methicillin-sensitive *Staphylococcus aureus* (MSSA) showed that patients with MSSA hematogenous vertebral osteomyelitis were more likely to have epidural abscess, and CRP values were higher in MSSA infection [6].

The overall mortality rate of our HPS patients at the 2-year follow-up was 14.8%, which is similar to rates from previous studies which ranged from 1.5 to 38% [1, 4, 6, 20, 21]. The large variance in these reported mortality rates may be attributed to different follow-up periods, varying in-hospital 6-month or 1-year mortality rates, and different causative microorganisms such as drug-resistant bacteria [6, 20, 21]. Park et al. reported an in-hospital mortality rate of 10%, and the overall mortality rate was 20% within 1-year follow-up [22]. Kehrer et al. found that the mortality remained high in the first year after admission [23].

To date, there are no evidence-based guidelines addressing the best treatment methods in the management of pyogenic spondylodiscitis [24]. In review of literature, the recommended duration of antibiotic therapy is from 6 weeks to 3 months, including a 2–8 week course of intravenous antibiotics and a 6 week-3 month course of oral antibiotics [24–26]. Although the mainstay of treatment for spondylodiscitis is long-term antibiotic therapy [25], surgical intervention is recommended in cases of spinal instability with vertebral destruction, abscess formation encroaching on psoas muscle or epidural spinal canal, and neurologic deficits.

Our study had several limitations including the small number of patients. Only a few patients were evaluated because we restricted HPS to a definitive diagnosis based on a microorganism isolated from the involved vertebrae in order to avoid the negative impact of misdiagnosis on the results. In addition, all patient data were collected from the Spine Operation Registry and HPS patients who received only antibiotic therapy were not included in this study. Combined antibiotic and surgical treatment was usually recommended in cases of deteriorated vertebral pathologic destruction, paraspinal abscess formation, and progressive sepsis. Although the characteristics of our patients may not be fully representative of all patients with HPS, the clinical features of patients in this study were consistent with those reported in the cohort studies of pyogenic spondylodiscitis, irrespective of surgical treatment [6].

## Conclusions

GNB-HPS patients were more likely to have a higher prevalence of malignancy, and GPC-HPS patient had a higher incidence of epidural abscess. GNB-HPS patients had more-frequent constitutional symptoms and GPC-HPS patients had predominant back pain. The clinical outcomes of GNB-HPS patients under combined surgical and antibiotic treatment were not different from those of GPC-HPS patients.

## Abbreviations

CRP: C-reactive protein
GNB: Gram-negative bacilli
GNB-HPS: Gram-negative bacillary hematogenous pyogenic spondylodiscitis
GPC: Gram-positive cocci
GPC-HPS: Gram-positive coccal hematogenous pyogenic spondylodiscitis
HPS: Hematogenous pyogenic spondylodiscitis
MRI: Magnetic resonance imaging
UTI: Urinary tract infection
